# Early blindness modulates haptic object recognition

**DOI:** 10.3389/fnhum.2022.941593

**Published:** 2022-09-08

**Authors:** Fabrizio Leo, Monica Gori, Alessandra Sciutti

**Affiliations:** ^1^Cognitive Architecture for Collaborative Technologies Unit, Istituto Italiano di Tecnologia, Genova, Italy; ^2^Unit for Visually Impaired People, Istituto Italiano di Tecnologia, Genova, Italy

**Keywords:** haptics, object recognition, blindness, exploration strategies, perception and action

## Abstract

Haptic object recognition is usually an efficient process although slower and less accurate than its visual counterpart. The early loss of vision imposes a greater reliance on haptic perception for recognition compared to the sighted. Therefore, we may expect that congenitally blind persons could recognize objects through touch more quickly and accurately than late blind or sighted people. However, the literature provided mixed results. Furthermore, most of the studies on haptic object recognition focused on performance, devoting little attention to the exploration procedures that conducted to that performance. In this study, we used iCube, an instrumented cube recording its orientation in space as well as the location of the points of contact on its faces. Three groups of congenitally blind, late blind and age and gender-matched blindfolded sighted participants were asked to explore the cube faces where little pins were positioned in varying number. Participants were required to explore the cube twice, reporting whether the cube was the same or it differed in pins disposition. Results showed that recognition accuracy was not modulated by the level of visual ability. However, congenitally blind touched more cells simultaneously while exploring the faces and changed more the pattern of touched cells from one recording sample to the next than late blind and sighted. Furthermore, the number of simultaneously touched cells negatively correlated with exploration duration. These findings indicate that early blindness shapes haptic exploration of objects that can be held in hands.

## Introduction

Humans can visually recognize objects in complex scenes in about one-tenth of a second (Potter, 1976; Thorpe et al., 1996). However, objects recognition is not a prerogative of vision. For instance, we can accurately identify real objects using only touch, although with a slower recognition time, in the order of seconds (Klatzky et al., 1985). The difference in recognition time between vision and touch is also due to intrinsic differences between the two sensory systems. Vision is usually characterized by holistic acquisition of information, whereas, touch often encodes information in a more sequential, and slower, fashion (Cattaneo and Vecchi, 2008). For instance, vision can decode simultaneously attributes of objects such as color and shape whereas touch may need different exploratory procedures, applied in sequence, to detect object properties such as texture and shape. We use indeed lateral motion to assess texture and contour-following to identify the shape (Lederman and Klatzky, 1987; Klatzky and Lederman, 1992). Visual and haptic object perception also differs for the weight they assign to different object properties (Lacey and Sathian, 2014). For instance, shape is more important than texture when visually categorizing, whereas shape and texture are approximately equally weighted in haptic categorization (Cooke et al., 2007).

However, visual and haptic object perception also shares some properties. For example, when considering object categorization, both vision and haptics show categorical perception, i.e., discriminability increases markedly when objects belong to different categories and decrease when they belong to the same category (Gaißert et al., 2012). In addition, both sensory modalities seem to be viewpoint-specific, i.e., they best recognize an object when it is oriented in a specific way although vision prefers “front-view” and haptics prefer “back-view” orientation (Newell et al., 2001).

Scientific works support the idea that these similarities may also have a neurophysiological foundation. Indeed, the visual and tactile sensory systems share some analogies also at the neural level (Amedi et al., 2005). They are both characterized by a hierarchical organization of increasing complexity. For instance, the unspecific tactile input is firstly processed in areas 3b and 1 of the primary somatosensory cortex, then by area 2 which shows selectivity to attributes of objects such as curvature and, finally, by the anterior intraparietal sulcus (IPS), which shows preference to overall shape rather than primitive attributes such as curvature (Bodegård et al., 2001). Both visual and tactile sensory systems show a topographical organization, i.e., adjacent parts of the space are mapped in adjacent parts in retinotopic and somatotopic cortical maps. More importantly, vision and touch may activate similar brain areas when exploring objects, for example, the visual ventral and dorsal pathways are also involved during similar haptic tasks (Amedi et al., 2005; Lacey and Sathian, 2011). For instance, James et al. (2002) found that haptic object exploration activated the middle and lateral occipital areas active in the corresponding visual exploration task. These cortical areas may be part of a network of neural substrates responsible for a supramodal representation of spatial information (Cattaneo and Vecchi, 2008; Loomis et al., 2013; Ottink et al., 2021). The existence of such supramodal representation is also suggested by other findings. For instance, Giudice et al. (2011) showed similar biases and updating performance when learning visual or tactile maps.

One might wonder what happens when the visual cortex does not receive visual input, as in blindness. It has been shown how the visual cortex can be functionally reprogramed in the blind to process tactile [see Sathian and Stilla (2010) for a review] or auditory stimuli (Kujala et al., 1995; Burton, 2003; Campus et al., 2019). As a consequence, the overall cortical representation of the tactile sense may be larger in the blind relative to sighted persons which may help explaining some superior tactile abilities, such as the higher tactile acuity, in the former population (Penfield and Boldrey, 1937; Goldreich and Kanics, 2003; Bliss et al., 2004; Wan et al., 2010; Norman and Bartholomew, 2011; Wong et al., 2011). However, haptic object recognition is a complex skill involving not only low-level tactile processing but also motor, memory, and spatial components. In particular, it has been suggested that visual mediation, that is, the translation of the tactile input into a visual image, may enhance haptic object recognition (Lederman et al., 1990). Therefore, according to the visual mediation hypothesis, we may hypothesize that object recognition based only on haptics may be superior in the late blind relative to congenitally blind or blindfolded sighted controls. Late blind individuals may indeed benefit of both extended haptic practice and the ability to translate the haptic information into a visual representation since they had seen earlier in life. Other researchers suggested that visual mediation may conduct to another advantage, that is the ability to represent spatial information in allocentric perspective. With allocentric representation we mean the ability to code spatial information based on an external perspective, independent from the observer, whereas, a representation is egocentric when it is based on the perspective of the observer (Taylor and Tversky, 1992). Allocentric representations are usually associated with higher spatial performance (Lawton, 1994; Meneghetti et al., 2011). It has been shown how blind individuals might prefer egocentric representations of spatial information while sighted persons tend to code the same information as allocentric, at least in the context of learning maps of environments (e.g., Noordzij et al., 2006). Toroj and Szubielska (2011) applied this framework to explain why their late blind participants, using an allocentric strategy when visualizing object shapes in their imagery, better identified such shapes than congenitally blind. The differentiation between egocentric and allocentric leads to the hypothesis that object recognition may depend also on the orientation of the objects relative to the participant. For instance, it has been shown how object recognition is impaired when the object is rotated with respect to the orientation of the learning phase which may be interpreted with the difficulty of moving from an egocentric to an allocentric perspective. This performance degradation is visible in the sighted regardless of the sense involved in recognition, that is vision or touch (Lacey et al., 2007). On the contrary, Occelli et al. (2016) showed that in the congenitally blind object recognition is view-independent, that is accuracy is not affected by the rotation of the learned object. Another result of this study is that overall no difference in performance between blind and sighted was observed. Szubielska and Zabielska-Mendyk (2018) also found similar ability in mentally rotating tactile figures in congenitally blind and sighted individuals.

Another line of research used two dimensional depictions of 3D shapes presented on raised line drawings. Using this kind of material, Heller (1989) found better recognition performance in late blind compared to sighted or congenitally blind persons. These latter two groups showed similar performance. On the contrary, Lederman et al. (1990) found that congenitally blind did worse than sighted in haptic recognition of 3D shapes and Gori et al. (2010) showed that congenitally blind children had higher orientation discrimination threshold compared to age matched controls. Collectively, these findings have been interpreted in terms of the necessity to visually translate the haptic information. In this perspective, the better performance in late blind may be the result of two factors: (1) their well-trained tactile skills; (2) their possibility to visually translate haptic information thanks to the fact they had seen earlier in life. This latter hypothesis is also well in line with a previous finding showing how the lack of visual experience in the early years of life can disrupt spatial processing in other sensory modalities (i.e., audition) suggesting the idea the visual system calibrates auditory spatial maps (Gori et al., 2014). However, the limited performance in early blind may not be present when manipulating real tridimensional objects. An early attempt to investigate this behavior in sighted and congenitally blind children has been performed by Morrongiello et al. (1994). The authors failed to find any difference in performance between the two populations. However, more recently, Norman and Bartholomew (2011) found even superior recognition accuracy of 3D shapes, not resembling daily-life objects, in early and late blind, but not in congenitally blind compared to sighted. Certainly, the contradiction between the studies may be due to the different tasks used and to possible differences in the tested populations.

In addition, to the best of our knowledge, most studies on this topic devoted little attention to the haptic patterns of exploration. For instance, studies using raised-lines drawings or textured pictures mainly focused on the final outcome in performance, that is recognition accuracy and time without investigating the haptic behavior conducting to that performance (e.g., Heller, 2002; Picard and Lebaz, 2012; Vinter et al., 2020). In Morrongiello et al. (1994), the authors also analyzed some basic haptic strategies of children exploring 3D objects. For instance, they measured the number of unique parts composing the object that was touched in a trial or the number of repetitions of exploration of those unique parts by examining video recordings. However, using this method, finer exploration features such as the number of touches of unique parts, their temporal frequency or the way subjects manipulated and rotated the objects could not be examined. Such haptic patterns may provide interesting complementary information as Leo et al. (2022) showed that different outcomes in performance in a haptic task may be associated with different haptic exploration strategies. Similarly, accuracy in haptic spatial tasks has been shown to depend on the level of development: children under 9 years of age showed indeed less effective haptic exploration than adults (Sciutti and Sandini, 2020). Furthermore, investigating such more detailed haptic exploration strategies may be necessary for identifying differences between groups of persons differing in spatial and visual ability. Therefore, in our study, we aimed at investigating: (1) how the performance in a haptic object recognition task is influenced by the level of visual ability; (2) how the level of visual ability shapes haptic exploration patterns. To do so, early blind, late blind, and sighted participants performed a haptic recognition task using an instrumented cube that measures the touches on its faces as well as its rotation, that is, the iCube (Sciutti and Sandini, 2019; Sciutti et al., 2019). As in Sciutti et al. (2019), we attached small pins on cube faces in varying number and asked participants to explore the cube twice, with the task of understanding whether any change occurred in the pins distribution between the first and the second presentation. This design is similar to a “study-test” paradigm to assess memory and recall (Pensky et al., 2008). Our study has a data-driven exploratory nature and several dependent variables recorded by iCube have never been collected in visually impaired subjects. However, we could at least expect that: (1) recognition accuracy may be similar across groups since the simple cube-like shape should not favor participants able to take advantage of a visual-mediation strategy; (2) both congenitally and late blind participants might be faster in doing the haptic task since they have larger haptic experience; (3) if it is true that blind persons and, particularly, congenitally blind prefer an egocentric representation of spatial information they might tend to rotate less the cube while exploring to facilitate the association of each cube face to its relative orientation.

## Materials and methods

### Participants

A group of congenitally blind (CB, *n* = 7, four females), a group of late blind (LB, *n* = 10, five females) and a sighted control group, age and gender matched with the visually impaired groups (SI, *n* = 16, nine females), took part in the study (see Table 1). One congenitally blind was excluded due to a technical issue with data collection. Following the World Health Organization (WHO) guidelines, we defined blindness as vision in a person’s best eye with correction of less than 20/500 or a visual field of less than 10°. All LB lose sight after 6 years of age. CB age ranged from 23 to 49 years (mean age = 35; SD = 9.5). LB age ranged from 30 to 61 years (mean age = 43.9; SD = 12). SI age ranged from 22 to 64 years (mean age = 40.7; SD = 12.1). Participants reported no conditions affecting tactile perception, or cognitive impairment. Blind participants were selected by the Istituto David Chiossone in Genoa and by the UVIP Unit of the Istituto Italiano di Tecnologia and agreed to participate on a voluntary basis. The experimental protocol was approved by the ethics committee of the local health service (Comitato Etico Regione Liguria, Genoa, Italy; Prot. IIT_UVIP_COMP_2019 N. 02/2020, 4 July 2020). All participants provided their written informed consent.

**TABLE 1:** Characteristics of the blind participants.

Participant	Gender	Age (years)	Etiology of visual impairment	Age at onset of complete blindness	Residual vision
**Congenitally blind**					
cb01	F	34	Retinopathy of prematurity	Birth	None
cb02	F	23	Retinopathy of prematurity	Birth	Light and shadow
cb03	M	32	Retinitis pigmentosa	Birth	None
cb04	M	29	Leber amaurosis	Birth	None
cb05	F	49	Retinopathy and glaucoma	Birth	Light and shadow
cb06	F	43	Atrophy optic nerve	Birth	None
**Late blind**					
lb01	M	34	Macular degeneration	20	Light and shadow, 1% visual field
lb02	F	56	Retinitis pigmentosa	35	Light and shadow
lb03	M	34	Corneal opacity	17	None
lb04	F	44	Accident, loss of retina	18	Light and shadow
lb05	F	61	Retinitis pigmentosa	40	Light and shadow
lb06	M	30	Leber amaurosis	19	Light and shadow
lb07	F	31	Optic nerve tumor	6	Light and shadow
lb08	F	61	Uveitis	11	None
lb09	M	45	Retinitis pigmentosa	34	Light and shadow
lb10	M	43	Retinitis pigmentosa	26	Light and shadow

### The iCube

**FIGURE 1:**
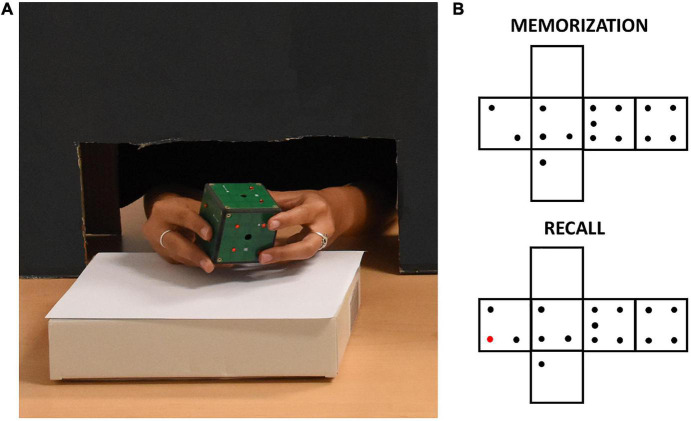
**(A)** A participant exploring the iCube with raised red pins positioned on its faces. The black cardboard panel avoided visual inspection of the device while allowing unconstrained haptic exploration. **(B)** Example of pins configurations of one trial. The red pin in the recall configuration indicates the difference with respect to the memorization configuration.

The iCube (v3) is an instrumented cube designed at IIT which measures its orientation in space as well as the location of contacts on its faces. This information is conveyed wirelessly to a laptop. iCube is of about 5 cm side, it has 16 cells per face and a weight of about 150 g (see Figure 1). Touch sensing is based on a 4 × 4 array of Capacitive Button Controllers (CY8CMBR2016) developed by Cypress Semiconductor Corporation. These are based on Multi Touch technology, allowing detection of simultaneous touches and support up to 16 capacitive cells (6 mm × 6 mm × 0.6 mm), which could be organized in any geometrical format, e.g., in matrix form. Each face of iCube is made with one of these boards. Their sensitivity, i.e., the smallest increase in capacitance that could be detected clearly as a signal, is set to 0.3 pF to allow the device to sense contacts without the need to apply pressure. Spatial orientation of the cube is estimated by a Motion Processing Unit™ (MPU), a nine axes integrated device, combining a three axes MEMS gyro, a three axes MEMS accelerometer, a three axes MEMS magnetometer and Digital Motion Processor™ (DMP). The MPU combines information about acceleration, rotation and gravitational field in a single flow of data. Data from iCube are sent to a laptop through a serial protocol. The transmission is performed through a radio module NRF24L01 (Nordic Semiconductor, Trondheim, Norway). The firmware of the device is designed to maximize the speed of capture of information from the boards measuring touches. The acquisition is always as fast as possible: faster when least faces are touched simultaneously and slower when it needs to encode information from multiple faces. As a result, the average sampling rate of the device was about 5 Hz (i.e., one sample every 203 ± 113 ms, SD). As in Leo et al. (2022), data were subsequently interpolated to analyze the temporal evolution of exploration at a constant temporal rate. Data generated in this study was further analyzed in Python (Python Software Foundation) to extract the pattern of touches, the amount of iCube rotation and the speed of rotation (see Section “Data Analysis”).

### Procedure

The experimenter positioned on iCube faces a set of raised plastic pins (diameter: 0.3 cm, height: 0.2 cm). Each face contained from 0 to 5 pins with no limitation of the presence of two or more equal faces. The participant was seated in front of a table, where the iCube was positioned on a support. Whenever a sighted participant was tested, a cardboard panel was placed on the table between him/her and the cube to avoid any visual inspection of the device. To do so, a black curtain was also fixed to the lower part of the panel on the side of the participant. This panel allowed anyway comfortable movements of participants’ upper limbs (see Figure 1). Before the experiment, participants performed a familiarization phase. In this phase, they first explored the cube without pins for a few seconds to get acquainted with it. After that, they did two practice trials in which they familiarized themselves with the experimental task, i.e., they were asked to explore the cube twice trying to understand whether any change occurred in the pins allocation between the first (memorization) and the second exploration (recall). Particularly, they were asked to report whether the cube in the second exploration was the “same” or “different” compared to the cube in the first exploration. When participants had proven to understand the task, the real experiment began. They did three trials in sequence for a total of six cube explorations for each participant. Between the memorization and recall phases, the cube could remain the same, but rotated on the support, or could be changed (e.g., by removing or adding one pin to one of the faces, see Figure 1 for an example). The experimenter rapidly operated these changes, with an interval between explorations lasting on average less than a minute. We opted for two “different” and one “same” trial to minimize participants’ fatigue as the latter trial-type has been shown as more difficult in previous studies (Norman et al., 2004; Sciutti et al., 2019). The experiment lasted about 30 min on average, including explanations and cube preparation.

### Data analysis

Data about touches and rotations recorded by iCube were processed in Python following the methods used in Leo et al. (2022) and briefly described below.

#### Touches

The cube reported for each timestamp a tactile map, i.e., a list of 16 elements of zeros and ones, where one represents a touched cell. These tactile maps were independently interpolated at a constant rate of 0.2 s, i.e., a value close to the average sample rate of the device. We then spatiotemporally filtered the tactile maps to select the explorative touches, i.e., touches directly related to the exploration of a face to detect and count its pins, from the holding touches, i.e., touches that only reflect the holding or support of the device. This filter was based on simple matching coefficient (SMC: $\frac{n\,u\,m\,b\,e\,r\,o\,f\,m\,a\,t\,c\,h\,i\,n\,g\,a\,t\,t\,r\,i\,b\,u\,t\,e\,s}{n\,u\,m\,b\,e\,r\,o\,f\,a\,t\,t\,r\,i\,b\,u\,t\,e\,s}=\frac{M\,00+M\,11}{M\,00+M\,01+M\,10+M\,11}$) which is a measure of similarity of samples sets with scores between 0 and 1, where 1 indicates perfect similarity and 0 indicates perfect diversity. M_11_ is the total number of cells where sample 1 and sample 2 both have a value of 1 (active); M_01_ is the total number of cells where the status of sample 1 is 0 (inactive) and the status of sample 2 is 1 (active); M_10_ is the total number of cells where the status of sample 1 is 1 (active) and the status of sample 2 is 0 (inactive); M_00_ is the total number of cells where sample 1 and sample 2 both have a value of 0 (inactive). Then, as in Leo et al. (2022) we assumed that explorative touches were characterized by higher variability in space and time than holding touches. Holding touches, by definition, are indeed stable in time to allow a secure grasping and movement of objects. For instance, the lateral motion exploratory procedure often associated with active exploration of a surface’s tactile features such as texture is characterized by highly dynamic movement of the hand in contact with the object. This kind of movement would translate for our sensors in a rapid change of status of cells activation in a face, resulting in lower SMC for consecutive temporal samples. Therefore, at each time interval we only considered explorative touches those measured on the face with the lowest SMC computed concerning the previous sample. If more than one face shared the lowest SMC, we considered the touches of all those faces, unless the SMC was 1 for all faces which would likely indicate the cube lying untouched on the table. We then computed the mean SMC of the explored faces for each trial. We used this variable as an indirect measure of velocity in exploring a face since, for instance, a very low SMC between two consecutive samples (0.2 s duration each) means that the participant touched very different cells between the two samples. We also computed: (1) the exploration duration of each trial as the time between the first and last touch of the participant (via manual cutting for each file the initial and final phases of recording, when less than two cells were active); (2) the mean exploration duration for each face; (3) the variability (i.e., standard deviation) of the mean exploration duration for each face; (4) the touch frequency, i.e., the number of touches per time unit (s); (5) the mean number of active cells per sample in the explored faces (after removing samples with no active cells).

#### Rotations

The information about the orientation of iCube in time was provided in the form of quaternions. Quaternions were interpolated at a constant sample rate of 0.2 s *via* spherical linear interpolation (SLERP). Then, we computed the instantaneous angular variation by measuring the angle traversed over time by each of the three unitary axes orthogonal to the faces of iCube. In particular, given one axis:


(1) $$\Delta \,{\text{angle}}_{\text{axis}}\,\left(\text{t}\right)=\text{arctan}\,\left(\left|\frac{a\,x\,i\,s\,\left(t\right)\,x\,a\,x\,i\,s\,\left(t-1\right)}{a\,x\,i\,s\,\left(t\right)\cdot a\,x\,i\,s\,\left(t-1\right)}\right|\right)*180^{{}^{\circ}}/\pi$$

We integrated over time the rotations performed by the three axes to estimate the rotation impressed to iCube in all the possible directions. To quantify the amount of rotation, we considered the maximum value among cumulative sums of the rotations executed by the three axes. The instantaneous rotation speed was instead computed by dividing Δangle_axis_(t) for its time interval (i.e., 0.2 s) and averaging the results across the three axes and all the instants in a trial in which iCube was in motion (i.e., angular velocity > 1°/s). As in Sciutti et al. (2019), this selection was made to assess the actual velocity of rotation when the rotations were executed, without spuriously reducing the estimate with the analysis of the static phases. In addition, we determined for each timepoint the absolute and relative orientation of each face of iCube. With absolute orientation we mean the cardinal direction of the normal of a face (with labels such as “North,” “East,” etc.). With relative orientation of a face we mean its orientation in the participant’s perspective (with labels such as “up,” “rear,” etc.). See Leo et al. (2022) for more details about these estimations.

#### Transition matrices

We computed the transition matrices for all the trials of the experiment, i.e., six by six matrices in which each cell corresponds to the percentage of cases in which the transition has occurred between the face individuated by the row number and the face corresponding to the column number (for instance, from “front” to “left”). Each trial is indeed characterized by a temporal sequence of explored faces (e.g., left, up, front, left, etc.). The transition matrix is computed by counting and summing the number of transitions (e.g., from “left” to “up”) and converting these numbers into percentage of occurrences. In particular, we computed a transition matrix for each trial in each participant (i.e., three matrices for the “memorization” trial type and three matrices for the “recall” trial type). Then, for each transition matrix we computed two different scores (Leo et al., 2022): (1) the maximum diagonal score; (2) the mean number of different transitions. The maximum diagonal score is the highest value in the diagonal cells. These cells reflect the tendency to select specific relative orientations as objects of spatial attention (e.g., a high proportion in the “from right to right” cell indicates that participant preferentially explored the rightward face and rotated the cube to position the face they wanted to explore toward their right). The number of different transitions is a measure of exploration variability (e.g., low numbers indicate participants selected less orientations to explore, i.e., less variability). For instance, a participant with a high maximum diagonal score and a low number of different transitions would be characterized by a very focused and systematic exploration reflecting high spatial ability (Leo et al., 2022). Finally, we measured the number of returns to already explored faces. For this measure, we did not consider the sequence of explored orientations but the sequence of explored faces in terms of their label (from 1 to 6). This measure may be relevant because a previous study showed that participants with lower spatial skill showed also an higher number of returns (Leo et al., 2022).

### Statistical analyses

Statistical analyses were performed using R. To sum up, we analyzed the following dependent variables: (1) recognition accuracy; (2) exploration duration (in s); (3) number of touches; (4) touch frequency (touches/s); (5) amount of rotation (°); (6) rotation velocity (°/s); (7) maximum diagonal score; (8) number of different transitions; (9) exploration duration per face; (10) variability of exploration duration per face; (11) number of returns; (12) mean number of active cells per sample; (13) mean SMC. The independent variables were the Group (early-blind, late-blind, sighted) and Trial Type (memorization vs. recall). Since we did not have specific hypotheses regarding the interaction between Group and Trial Type and since the comparison between memorization and recall in the same task has been already investigated in Sciutti et al. (2019) we only focused on group differences. Given the high number of dependent variables we ran an explorative MANOVA including all the normally distributed dependent variables (all but recognition accuracy) with Group as between factor. For recognition accuracy, after a Box-Cox transformation using the MASS R package (Venables and Ripley, 2002), we estimated a Bayes factor to compare the fit of the data under the null hypothesis and the alternative hypothesis using BayesFactor R package (Morey and Rouder, 2011). Data normality was assessed with Shapiro-Wilk tests. After the MANOVA we also performed a Linear Discriminant Analysis (LDA) as follow-up with the goal of defining which linear combination of dependent variables led to maximal group separability. We then conducted univariate ANOVA on the dependent variables that showed higher coefficients in the LDA followed by *t*-tests as *post hoc*. We corrected for multiple comparisons using Benjamini/Hochberg FDR correction (Benjamini and Hochberg, 1995a,b). We set statistical significance at *p* < 0.05.

## Results

As for the iCube recognition, the mean accuracy was 72% for the CB, 77% for the LB and 69% for the SI. The estimated Bayes factor suggested that the data were 3.7 times more likely to occur under a model without including an effect of group, rather than a model with it.

The MANOVA revealed a significant difference between groups in the haptic exploration variables [Roy (2,12) = 1.84, *p* = 0.018].

**FIGURE 2:**
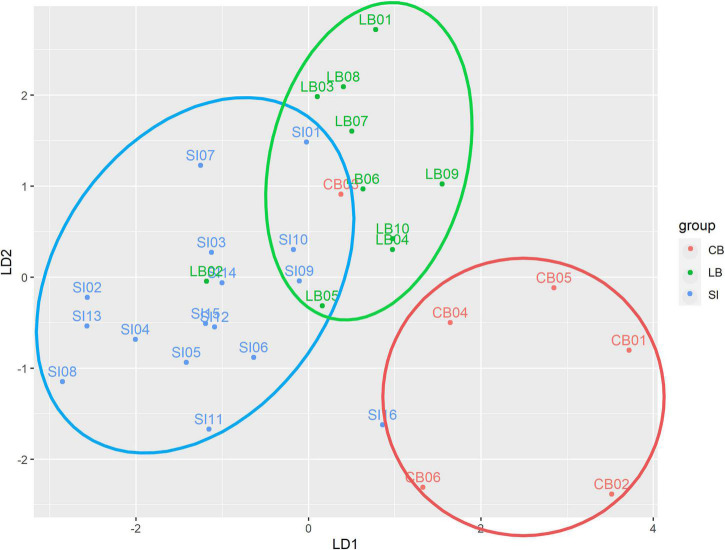
Scatterplot of participants distribution in the two LDA dimensions. The diagram depicts congenitally blind (CB) as red circles, late blind (LB) as green circles and sighted controls (SI) as blue circles. The labels above each circle specify participants’ code. Ellipses indicate the three identified clusters. Note as the three groups tend to concentrate in different areas of the 2D space as defined by the two discriminants.

The follow-up LDA identified two linear discriminants which accounted for a percentage of separation between groups of 74.8 and 25.2%, respectively. The haptic variables which were able to discriminate more strongly the groups were the mean SMC, the mean active cells per sample and the maximum diagonal score. Table 2 shows the normalized coefficients of linear discriminants. Figure 2 shows participants distribution along the two discriminants. It is evident how the three groups concentrate in different areas defined by the two discriminants. Both CB and LB participants tend to have higher scores than SI in LD1. As for the LD2, while SI showed intermediate levels, LB and CB showed higher and lower scores, respectively. Finally, CB tend to form a quite separate cluster whereas LB and SI clusters show higher superposition.

**TABLE 2:** Coefficients of linear discriminants (LDA).

Haptic variable	LD1	LD2
Exploration duration	0.09	0.06
Number of touches	–0.02	–0.01
Touch frequency	–0.42	0.59
Amount of rotation	0.00	0.00
Rotation velocity	–0.07	0.00
**Maximum diagonal score**	**6.56**	**–1.20**
Number of different transitions	0.48	0.12
Exploration duration per face	–0.45	–0.69
Variability in exploration duration per face	0.49	0.87
Number of returns	–0.18	0.34
**Mean active cells per sample**	**3.17**	**–1.44**
**Mean SMC**	**−9.36**	**15.51**

Coefficients for each linear discriminant. Bold indicates haptic variables whose linear combination discriminated more strongly between groups (absolute value > 1).

**FIGURE 3:**
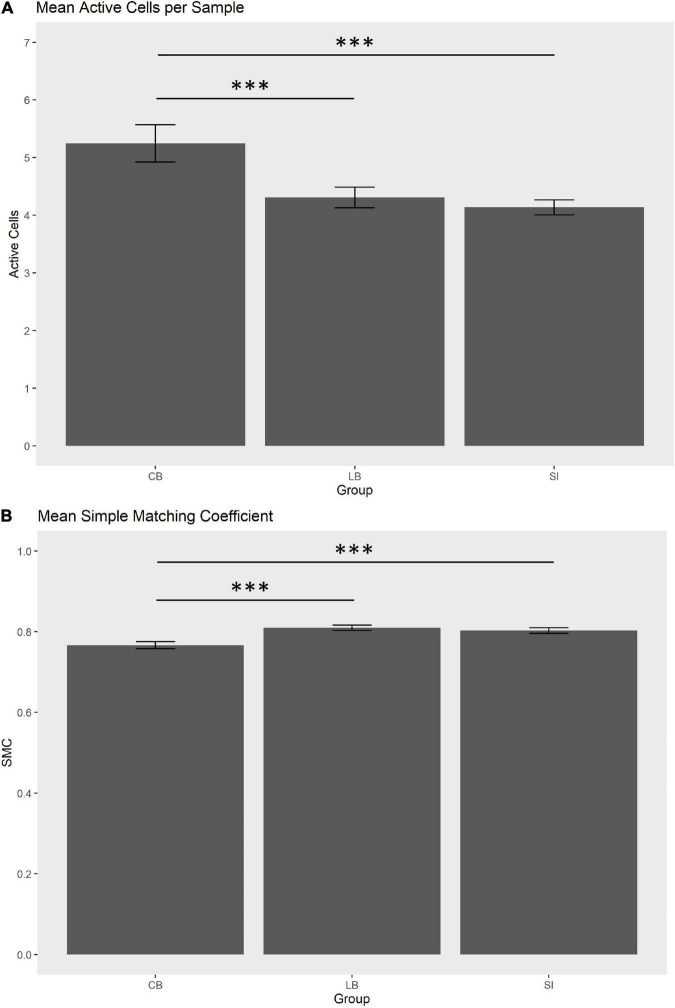
**(A)** Mean active cells per sample (0.2 s) in the explored face. **(B)** Mean simple matching coefficient (SMC) in the explored face. Whiskers indicate SEM. ***p_fdr_ < 0.001.

In order to statistically substantiate these differences, we ran a one-way ANOVA for each of the three haptic variables that contributed more in discriminating the groups, i.e., max diagonal score, mean SMC and mean active cells per sample. As for the maximum diagonal score, the groups did not differ [CB = 3.41, LB = 3.27, SI = 2.24; *F*_(2,29)_ = 0.87, *p* = 0.43]. As for the mean active cells per sample in the explored face, the groups tend to differ [CB = 5.24, LB = 4.32, SI = 4.14; *F*_(2,29)_ = 3.75, p_*unc*_ = 0.035, p_fdr_ = 0.07]. *Post hoc* tests showed that the number of active sensors was higher in the CB than in the SI [*t*_(44.8)_ = –4.96, p_fdr_ < 0.001; see Figure 3A] and in the LB [*t*_(55.5)_ = 3.91, p_fdr_ = 0.00038; see Figure 3A]. The comparison between SI and LB was not significant (*p* = 0.22). As for the mean SMC, this score tend to differ in the three groups [CB = 0.77, LB = 0.81, SI = 0.80; *F*_(2,29)_ = 3.38, p_unc_ = 0.047, p_fdr_ = 0.07; see Figure 3B] since it was lower in the CB than in the SI [*t*_(59.6)_ = 4.14, p_fdr_ = 0.00017] and in LB [*t*_(68.6)_ = –4.31, p_fdr_ = 0.00016]. No difference was observed between LB and SI (*p* = 0.58).

**FIGURE 4:**
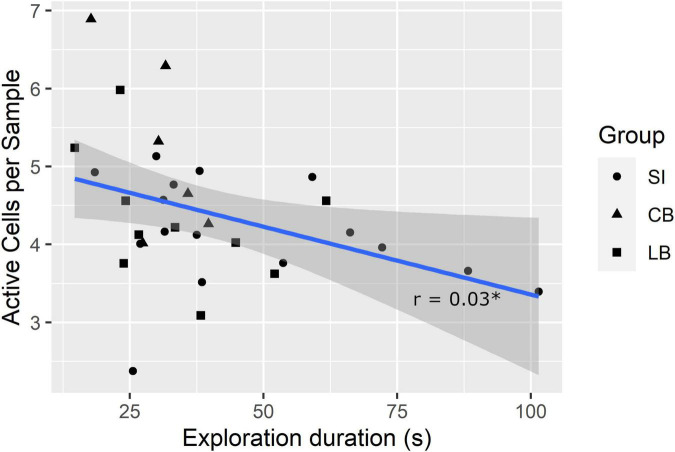
Correlation between exploration duration and mean number of active cells per sample. *p_fdr_** <** 0.05.

A lower SMC and higher mean number of active cells per sample in the explored faces are potentially indexes of faster exploration because the former indicates the participant considerably changed the touched cells from one sample to the next and the latter shows that more cells were simultaneously considered. Therefore, we further hypothesized that SMC score and number of active cells per sample would correlate positively and negatively, respectively, with exploration duration. To verify these hypotheses, we computed Pearson’s correlation coefficients (r). Results showed that the SMC did not correlate with exploration duration (*r* = 0.21, *p* = 0.127, one-tailed), whereas, the number of active cells per sample did (r = –0.38, p_fdr_ = 0.03, one-tailed; see Figure 4).

## Discussion

Our study had two different aims: first, investigating whether the level of visual ability modulates haptic object recognition; second, highlighting possible differences in the exploration strategies in congenitally blind, late blind, and sighted individuals using a sensorized cube. To do so, we asked a group of congenitally blind, a group of late blind and a group of sighted persons (who could not see the device) to explore twice an iCube with pins attached to its faces. In the second exploration, the iCube could have the same pins disposition, although the cube would be presented in a different orientation, or a small change in pins disposition, e.g., one pin less or more in one of the faces. Participants had to report whether the two presented cubes had the same pin disposition, or they differed. The main advantage of using the iCube compared to common daily-life objects lies in that it allows a free and unconstrained manipulation while keeping the possibility of accurately measuring how it is touched and its orientation in space without the need to use video recordings.

Our results showed that the level of visual ability does not influence the accuracy in recognizing the cube. This finding is in line with Morrongiello et al. (1994), who, in addition, also failed to observe differences between blind and sighted children in terms of exploration behavior. However, in our case, we showed evidence of different haptic strategies between congenitally blind and the other groups. Indeed, congenitally blind tend to touch simultaneously more cells in each recording sample when exploring a face than late blind and sighted persons, suggesting that they learnt to consider a larger tactile space with a single touch. They also tend to change touched cells more quickly than the other groups. This is an important result because it suggests that congenitally blind persons may have a peculiar way to explore the environment through touch, which differentiates them even from late blind persons characterized by many years of complete blindness, as in our sample of participants. Furthermore, we observed that the number of simultaneously touched cells negatively correlated with exploration duration. If we can cover a larger tactile space with a single touch, then the time needed to fully explore an object decreases. It should be noted that a previous study showed evidence of an impairment in haptic recognition of faces in the congenitally blind and not in late blind suggesting that early visual experience is necessary to process face features (Wallraven and Dopjans, 2013). However, there is also evidence that faces may be special kind of “objects” processed by dedicated brain areas in the human visual system, such as the fusiform gyrus (Puce et al., 1995; Yue et al., 2006). Therefore, findings on faces recognition in the blind may not be easily translated to different types of objects.

Our third hypothesis, i.e., blind participants would rotate less the cube was not supported by results. However, this may simply be due to the reduced power of our analysis since congenitally blind and late blind tended to rotate less the device (560° and 517°, respectively) than sighted (710°).

Importantly, our findings do not seem to be due to differences in spatial memory in the groups of participants. There is evidence that congenitally blind subjects may have difficulties in specific spatial memory tasks, particularly when they have to memorize and recall two separate haptic spatial configurations (Vecchi et al., 2004; Leo et al., 2018, 2020) or sequences of semantic sounds. However, in our study the congenitally blind showed a similar recalling accuracy than the other groups. Our task did not impose indeed a heavy burden on spatial memory since participants were required to keep in memory only five items (the number of pins in five faces) and their relative location. On the contrary, in Leo et al. (2018) participants had to memorize an average of 2.5 targets randomly located in a 3 × 3 grid and they had to do so for two different grids presented in sequence. This task is much more complex because there are many ways to place 2.5 targets in a nine-elements grid and participants had to keep in memory two of these grids.

The conflict between our and Morrongiello et al.’s (1994) findings who did not observe haptic differences in object recognition between blind and sighted participants may be due to several reasons: (a) Morrongiello and coauthors tested only children. It is possible that the differences we found in haptic patterns would emerge only later in life, as a consequence of the more extended haptic training [but see Withagen et al. (2012) for a similar result with adults]; (b) they used common daily-life objects, whereas we used two cubes eventually differing between each other only for relative pin disposition on the surface of their faces; (c) they studied haptic behavior through evaluation of video recordings, that is with a methodology and a selection of dependent variables which may be not sensitive enough to detect subtle differences in exploration procedures.

On the other hand, there is also evidence in the literature regarding differences in exploratory procedures between blind and sighted children, although in studies using different materials and methods. For instance, Vinter et al. (2012) asked blind, low vision, and blindfolded sighted children to haptically explore raised-line drawings whose comprehension was subsequently evaluated through drawings of the remembered shapes. Briefly here, results showed how blind children used more types of exploratory procedures, as defined in Davidson (1972), Lederman and Klatzky (1987, 1993), and Wijntjes et al. (2008), than their sighted peers. The use of certain kinds of procedures (e.g., contour following) also correlated with drawing performance. However, this study referred to the classical exploratory procedures originated by the seminal work of Lederman and Klatzky (1987) which cannot easily be translated to the case of solid objects such as our cube.

While the fact that congenitally blind participants used different haptic strategies may be simply due to their higher training in using only the haptic modality, it is also possible that these differences could be partly due to divergent spatial strategies between congenitally blind, sighted and late blind persons. Previous studies suggested indeed that sighted individuals might prefer using an allocentric frame of reference (Noordzij et al., 2006; Pasqualotto et al., 2013) which, although accurate, may need more time to be built (Toroj and Szubielska, 2011). Even though we did not explicitly investigate this issue, two congenitally blind participants spontaneously reported they counted the number of pins of the cube faces to help memorizing pins configuration which suggests they were not using an allocentric strategy. This observation is also well in line with a previous finding showing that early blind subjects encoded 2D pattern elements by their location in a fixed coordinate system without visual representation (Vanlierde and Wanet-Defalque, 2004). Future studies might want to investigate in detail such cognitive aspects of haptic exploration using the iCube.

With our current data, it is difficult to conclude whether the difference between congenitally and late blind is due to the fact the former group has never experienced the visual world and, therefore, it has exploited the brain plasticity that strongly characterizes the early years of life (e.g., Kupers and Ptito, 2014) resulting in a stronger haptic ability (Theurel et al., 2013) or to the fact that haptic skills are simply more trained in the congenitally blind since they lived more “years of blindness.” Our congenitally blind group has experienced a mean of 35.5 years of blindness, whereas, this mean in the late blind group was 21.6 years. Future studies will be needed to compare exploration behavior of congenitally and late blind individuals having a similar amount of years of blindness (although, in this case, differing for age). On the other hand, we speculate that, since our late blind participants were probably fully blind for long enough to match the haptic expertise of the congenitally blind, the main difference between the two groups may lie in the extended haptic practice in the congenitally blind in their early years of life (Theurel et al., 2013; Amadeo et al., 2019).

One limitation of our study lies in the small sample size, particularly the congenitally blind group. This may have limited the possibility to spot other haptic differences between this group and late blind and sighted groups. However, specific differences between groups, that is, the mean number of active cells per sample and the variability in active cells across recording samples, were evidently large enough to be already detected with groups of such size. A second limitation lies in that information about Braille-reading ability in our blind participants was not available. There is evidence that experience in reading Braille is correlated with superior tactile acuity in passive tasks (Wong et al., 2011) and in tasks using Braille-like stimuli (e.g., Foulke and Warm, 1967; Grant et al., 2000). However, our task involved the active manipulation of a 3D object and the pins attached on its faces have different dimension (diameter: 3 mm; height: 2 mm) than Braille dots (diameter: 1.44 mm; height: 5 mm). More importantly, the spacing between pins in our configuration is in the order of centimeters whereas it is about 2.5 mm in the Braille. Therefore, our task did not involve any measure of tactile acuity at its limit of performance, as Wong et al. (2011) did. A third limitation is represented by the fact we used a cube-shaped object which imposes limits in the exploration behavior of participants and makes potentially difficult generalizing our results to objects with more complex shapes. Finally, subjects performed a small number of trials since we wanted to minimize the effort of participants. Therefore, we could not investigate in detail the temporal evolution of performance as well as possible changes in exploration strategies.

In conclusion, our study showed that congenitally, late blind and sighted participants did not differ in the haptic recognition accuracy of a three-dimensional object. However, we identified two exploratory strategies that differentiated congenitally blind from late blind and sighted individuals. The former group touched more cells simultaneously when exploring a face, suggesting that they could acquire more tactile information “at first glance.” Furthermore, congenitally blind showed higher haptic velocity, that is, they changed more the pattern of touched cells from one recording sample to the next. Finally, we also found that the number of simultaneously touched cells negatively correlated with exploration duration suggesting that the ability to cover a larger tactile space while touching an object allows a more effective and faster exploration.

Future studies might want to verify whether we could use the sensorized cube to measure the haptic and spatial skills of different populations such as in the elderly. There is indeed evidence that cognitive decline may impair haptic object recognition (Kalisch et al., 2012) but the modulation of the exploratory procedures by age has not been investigated in detail yet.

## Data availability statement

The datasets presented in this study can be found in online repositories. The names of the repository/repositories and accession number(s) can be found at: https://doi.org/10.5281/zenodo.6539275.

## Ethics statement

The studies involving human participants were reviewed and approved by Comitato Etico, ASL 3, Genova; Prot. IIT_UVIP_COMP_2019 N. 02/2020, 4 July 2020. The patients/participants provided their written informed consent to participate in this study. Written informed consent was obtained from the individual(s) for the publication of any potentially identifiable images or data included in this article.

## Author contributions

FL performed testing and data analysis. FL wrote this manuscript with contributions from MG and AS. All authors developed the study concept, contributed to the study design, and approved the final version of the manuscript for submission.
